# A Rat Model of the Brain-Derived Neurotrophic Factor Val66Met Polymorphism Shows Attenuated Motivation for Alcohol Self-Administration and Diminished Propensity for Cue-Induced Relapse in Females

**DOI:** 10.3390/biology12060799

**Published:** 2023-05-31

**Authors:** Emily J. Jaehne, Elizabeth McInerney, Ronan Sharma, Shannyn G. Genders, Elvan Djouma, Maarten van den Buuse

**Affiliations:** 1 Department of Psychology, Counselling and Therapy, School of Psychology and Public Health, La Trobe University, Melbourne 3086, Australia; e.jaehne@latrobe.edu.au (E.J.J.);; 2 Department of Microbiology, Anatomy, Physiology and Pharmacology, School of Agriculture, Biomedicine and Environment, La Trobe University, Melbourne 3086, Australiae.djouma@latrobe.edu.au (E.D.); 3 Department of Pharmacology, University of Melbourne, Melbourne 3052, Australia

**Keywords:** brain-derived neurotrophic factor, Val66Met, alcohol, operant, breakpoint, relapse

## Abstract

**Simple Summary:**

The growth factor brain-derived neurotrophic factor (BDNF) has been implicated in alcohol use disorder. Val66Met is a common variant of the BDNF gene which reduces BDNF release in the brain. Val66Met has been suggested as a risk factor for psychiatric disorders and substance use. Using an operant self-administration paradigm, we investigated ethanol preference and ethanol seeking in a genetically modified rat model of the BDNF Val66Met variant, Val68Met rats. There was no effect of Val68Met genotype (Val/Val, Val/Met and Met/Met) on acquisition of stable lever pressing for a 10% ethanol solution or extinction of this behaviour. Met/Met rats of both sexes had slightly, but significantly lower motivation to lever press for ethanol. Following extinction of responding, females with the Met/Met genotype demonstrated a lower propensity for reinstatement of responding to cues. There were no changes in anxiety-like behaviour or locomotor activity. In conclusion, Met/Met rats showed lower motivation to press for a reward, and also a decreased propensity to relapse, suggesting a possible protective effect of the Met/Met genotype against alcohol use disorder, at least in females.

**Abstract:**

Brain-derived neurotrophic factor (BDNF) has been implicated in alcohol use disorder. The Val66Met polymorphism is a common variant of the BDNF gene (rs6265) which reduces activity-dependent BDNF release, and has been suggested as a risk factor for psychiatric disorders and substance use. Using an operant self-administration paradigm, this study aimed to investigate ethanol preference and ethanol seeking in a novel rat model of the BDNF Val66Met polymorphism, Val68Met rats. Male and female BDNF Val68Met rats of three genotypes (Val/Val, Val/Met and Met/Met) were trained to lever press for a 10% ethanol solution. There was no effect of Val68Met genotype on acquisition of stable response to ethanol or its extinction. Met/Met rats of both sexes had a slight, but significantly lower breakpoint during progressive ratio sessions while female rats with the Met/Met genotype demonstrated a lower propensity for reinstatement of responding to cues. There were no effects of Val68Met genotype on anxiety-like behaviour or locomotor activity. In conclusion, Met/Met rats showed lower motivation to continue to press for a reward, and also a decreased propensity to relapse, suggesting a possible protective effect of the Met/Met genotype against alcohol use disorder, at least in females.

## 1. Introduction

Alcohol use disorder (AUD) is characterised by uncontrollable consumption of alcohol for a long period of time, to the degree that daily living is impacted [1]. Lifetime AUD is prevalent in 29.1% of the world’s population [2], and chronic alcohol abuse is responsible for up to 3 million deaths annually [3]. Relapse rates following abstinence are high [4] and appear to be influenced by a range of biological and psychosocial factors [4,5,6]. Despite a wide array of research on alcohol’s prevalence and problematic impacts, many questions remain as to why some individuals are more susceptible to AUD and relapse than others [7].

Brain-derived neurotrophic factor (BDNF) has a demonstrated role in the body’s response to alcohol [8,9]. For example, alcohol-preferring rats had lower BDNF levels in the amygdala and other brain areas than non-preferring controls [10]. Injection of BDNF into the ventral tegmental area (VTA) reduced excessive ethanol consumption [11]. BDNF expression in the medial prefrontal cortex (mPFC) [12,13] and the dorsolateral striatum [14] have also been associated with increased alcohol consumption [7,15]. A protective role of BDNF is furthermore suggested by findings of higher preference for ethanol among mice with BDNF deficiencies. BDNF heterozygous (HET) mice showed higher baseline ethanol consumption, and higher relapse rates when compared to wildtype controls [16,17,18]. Sex differences in the involvement of BDNF in alcohol responding have also been described. In an operant ethanol self-administration paradigm, female, but not male BDNF HET rats demonstrated a greater propensity to reinstate their lever press responding after an extinction period, compared to wildtype controls [19].

Given these results, BDNF mimetics would be predicted to be useful in treating alcohol seeking and relapse. Indeed, in a model of intermittent access, two-bottle choice (IA2BC) ethanol consumption in rats, systemic administration of the flavonoid, 7,8-dihydroxyflavone (DHF), which mimics the physiological functions of BDNF, reduced excessive ethanol consumption and preference [11]. On the other hand, in an operant ethanol self-administration paradigm, subchronic administration of DHF enhanced reinstatement in rats [20]. These and other findings [8,21] suggest that the role of BDNF in AUD may vary between different aspects of the disorder.

These preclinical studies predict differences in human subpopulations depending on genetic changes in BDNF signalling [8]. The BDNF gene variant Val66Met leads to reduced activity-dependent release of BDNF in the brain [22,23]. Although two meta analyses of gene association studies failed to find an association of BDNF rs6265 with alcohol dependence [24,25], other studies have suggested links between Val66Met and alcohol intake [7,26,27,28]. Clinical studies have suggested that users with the Val/Val genotype show shorter time intervals between abstinence and relapse, and higher relapse rates in comparison to Met allele carriers [29]. Using a IA2BC paradigm in a Val68Met transgenic mouse model, Warnault et al. [30] observed that Met/Met mice consumed excessive amounts of alcohol compared with the wild-type mice. On the other hand, in an operant ethanol self-administration protocol using Val66Met mice, females with the Val/Val genotype showed greater ethanol seeking than Val/Val males, but there were no sex differences in Met/Met mice [31]. Female Val/Val mice also showed greater impulsivity than female Met/Met mice but there were no genotype differences in breakpoint during operant progressive ratio sessions [31]. These preclinical and clinical findings show that the role of BDNF in AUD remains unclear and could differ between different stages of alcohol use and different experimental models [32].

Using an operant lever press paradigm, this study aims to investigate different aspects of ethanol preference and voluntary alcohol seeking in a novel rat model of the BDNF Val66Met polymorphism, Val68Met rats, which show the expected reduction of activity-dependent BDNF release [33]. We compared males and females of three genotypes (Val/Val, Val/Met, or Met/Met). Additional behavioural testing was also conducted to ensure any potential differences in alcohol responding were not a result of effects of genotype on anxiety or locomotor activity behaviours.

## 2. Materials and Methods

### 2.1. Animals and Housing

A total of 91 BDNF Val68Met rats on a Sprague–Dawley genetic background [33] were held and tested at the La Trobe Animal Research and Teaching Facility (LARTF). The animals were offspring from Val/Met breeding pairs derived from a breeding colony at the Australian Resources Centre (Perth, Australia). Six testing groups consisted of 11–17 animals of three genotypes (Val/Val, Val/Met, Met/Met) and two sexes (male and female) (Table 1; see Supplementary Figure S1 for body weights throughout the experimental period). No more than two rats of each sex per litter were used in each group to prevent the influence of litter effects.

All rats were housed in standard individually ventilated cages (IVC, Tecniplast, Bugaggiate, Italy) for the duration of the experiment (4/cage). The animals had ad libitum access to food and water and were housed under standard laboratory conditions (21 ± 2 °C, 55% ± 15% humidity) under a 12:12 h reverse light–dark cycle (lights off 7 a.m.). All behavioural testing was conducted during the dark phase of the light cycle between 9 a.m. and 3 p.m. under red light conditions.

All procedures were compliant with the Australia Code of Practice for the Care and Use of Animals for Scientific Purposes (National Health and Medical Research Council of Australia) and were approved by the La Trobe University Animal Experimentation Ethics Committee (AEC:21002).

### 2.2. Behavioural Testing

#### 2.2.1. Operant Protocol

**Figure 1:**
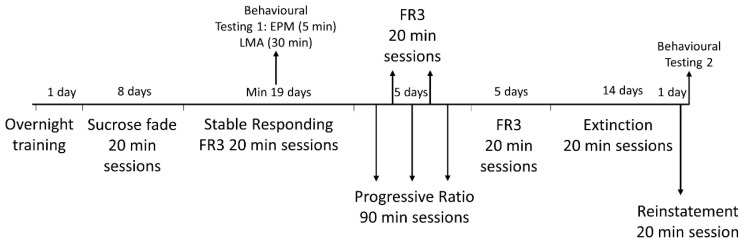
Experimental timeline: lever responding was first monitored during overnight training then sucrose fade, and continued through FR3, progressive ratio, extinction, and reinstatement. Behavioural testing was conducted halfway through the FR3 stable responding period and again following the completion of operant testing. FR3, fixed ratio of 3 presses per 1 reward; EPM, elevated plus maze; LMA, locomotor activity.

Self-administration sessions were carried out as per previous studies [20,31] in operant conditioning chambers (Med Associates, St. Albans, VT, USA) that were housed in sound-attenuating cubicles with a house fan to provide ventilation and mask external noises. An ultra-sensitive retractable stainless-steel lever was situated on both left and right walls of the chamber, one representing “active” (ethanol) and the other “inactive” (water). Each lever was linked to a fluid reservoir that would dispense 100 μL of solution into a receptacle once the fixed ratio (FR) of lever presses was achieved. A stimulus light was located next to each lever and would illuminate once the required FR lever presses were obtained. All chambers were linked to a computer running MED-PC IV software to configure the needed operant settings and record data. The operant protocol consisted of several consecutive stages (Figure 1).

Overnight Training: When approximately six weeks old, the rats were introduced to the operant chambers in a single overnight training session which lasted approximately 16 h, where rats were able to investigate the chamber and learn how to lever press for a single 100 μL reward of 5% *v*/*v* ethanol and 5% *v*/*v* sucrose or a water reward upon 2 lever presses (FR2). Rats were also familiarised with cues to signal reward availability. This included a stimulus light upon reward delivery and an olfactory cue of vanilla essence placed underneath the active lever. Animals had sufficient access to food throughout the night with standard rat chow available ad libitum for the entirety of the overnight training.

Sucrose Fade: Following overnight training, animals progressed to the sucrose fade protocol, where the concentration of sucrose was gradually reduced so that rats were eventually only responding for a solution of 10% *v*/*v* ethanol. Rats were placed in the operant chambers for daily 20 min sessions (Monday–Friday) for 8 days. A solution of 10% *v*/*v* ethanol and 5% *v*/*v* sucrose was administered on days 1–5, and 10% *v*/*v* ethanol, 2.5% sucrose from day 6–8. Ethanol and water levers were alternated on day 4 to avoid place preference. A drop of solution was placed in each receptacle and vanilla essence was placed before the active lever to signal the orientation of the solutions. A fixed ratio of FR2 was used on day 1, then increased to FR3 where it stayed for the duration of the sucrose fade.

FR3 Acquisition—Stable Responding: After 8 days of decreasing the sucrose concentration, rats progressed to a solution of 10% *v*/*v* ethanol, which was considered the reward for the continuation of the study. For the following 4 weeks, a fixed ratio of FR3 was set for the 20 min daily sessions. The olfactory cue of vanilla essence and the stimulus light was again paired with the presence of alcohol.

Progressive Ratio: Following normal responding, rats entered progressive ratio (PR), where the number of lever presses required for a single reward of either solution increased following each reward dispensed; for example, first reward following one lever press, second reward after three presses, third reward delivered after six presses. This was the case for both active and inactive levers. A total of three PR sessions were conducted for a duration of 90 min every second day for five days (Monday, Wednesday and Friday, see Figure 1), with two 20 min FR3 sessions in between (Tuesday and Thursday). Breakpoint was defined as the point where rats would cease pressing the lever the required number of times for a single reward of ethanol or water.

Extinction: Once completing breakpoint testing, an additional five days of FR3 was conducted before proceeding onto 3 weeks of extinction (Figure 1). Ethanol, water and vanilla essence were removed, and the stimulus light turned off for daily 20 min sessions, which subsequently resulted in rats gradually diminishing their propensity to lever press.

Reinstatement: Following extinction and establishing a baseline where ‘active’ lever responding was similar with ‘inactive’, the rats underwent a single 20 min reinstatement session where conditioned cues (vanilla essence and stimulus light) and a single drop of each solution in their respective receptacles were re-introduced. Importantly, during this session ethanol and water rewards were withheld upon lever presses. Rats were expected to revert to a preference for pressing the lever associated with ethanol reward over the lever associated with water. The number of lever presses during the reinstatement session were compared with those of the last 5 days of extinction.

#### 2.2.2. Elevated plus Maze

The elevated plus maze (EPM) test was conducted on two occasions throughout the operant protocol on a day when no operant responding was assessed. The first session, 5 min for each rat, was conducted approximately halfway through the FR3 phase, with the second session conducted several days following completion of the reinstatement session. The maze was raised approximately 50 cm above the floor and consisted of a plus shape with two 50 cm long open arms and two 50 cm long closed arms [34]. An infrared camera above the maze was used to record behaviour and Ethovision software (Noldus, Wageningen, The Netherlands) analysed rat movements including the time spent in each arm of the maze. A reduced amount of time spent exploring the open arms was taken to indicate more anxiety-like behaviour [34].

#### 2.2.3. Locomotor Activity

Locomotor activity was assessed in a 30 min session conducted immediately after each of the two elevated plus maze sessions and examined whether factors such as sedation or hyperactivity influenced lever pressing. Rats were tested in locomotor photocells (H:31 × W:43 × L:43 cm; MED Associates, St. Albans, VT, USA) that detected all movements across infrared beam arrays. Data were recorded using MED-PC IV software. Greater distance covered (cm) was a potential indicator of hyperactivity, while significantly lower locomotion suggested lethargy [34].

### 2.3. Data Analysis

Data analysis was carried out using SPSS version 27 (IBM Inc., Chicago, IL, USA) and GraphPad Prism version 9 (GraphPad Software, San Diego, CA, USA). Mixed between-within ANOVAs with sex and genotype as between-subject factors and lever and session as the repeated measures, followed by appropriate post hoc testing, was used to analyse all data. Data are expressed as the mean ± standard error of the mean (SEM), with *p* ≤ 0.05 regarded statistically significant.

Data were first checked for outliers in each stage of testing using z-scores, and scores falling outside z = ±3.29 were removed. Two additional values were excluded from the reinstatement session based on rats activating <2 rewards (five or fewer presses) over the 20 min period [20]. Data were then checked for normality violations using skewness and kurtosis z-scores and scores outside z = ±1.96 considered to be violating normality. ANOVA has been suggested to be robust to mild violations of normality given that the sample size is at least 30 [35]. If the assumption of sphericity of repeated-measures was violated a more conservative Greenhouse–Geisser degrees of freedom adjustment was used. For all main analyses, the null hypothesis was rejected at *p* < 0.05. A Bonferroni adjustment was used for all post hoc analyses where appropriate. Magnitude of effect sizes (*ηp*^2^ ≥ 0.01 small, ≥0.06 medium, and ≥0.14 large) was defined using guidelines from Cohen [36].

## 3. Results

### 3.1. Body Weight

**Figure 2:**
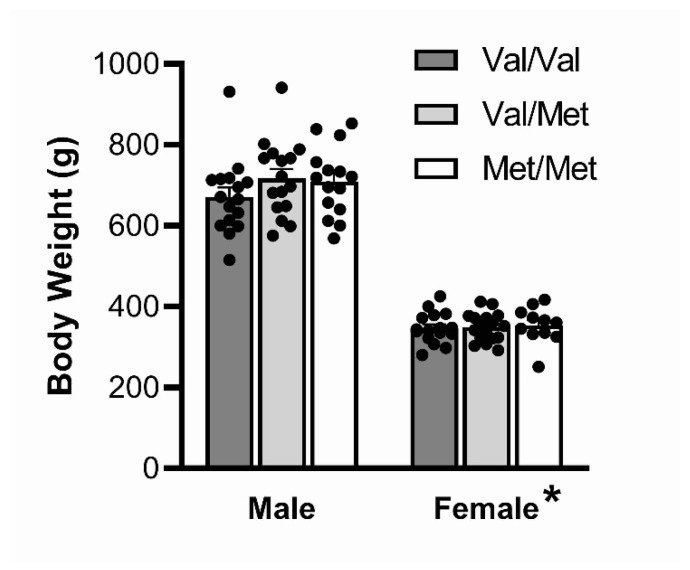
Final body weight. Males weighed significantly more than females at the end of the experiment; however there were no significant weight differences between genotypes. Data represent mean ± SEM (n = 11–16/group). * *p* < 0.001 compared to males.

Males weighed significantly more than females at the end of the experiment (F(1,85) = 544.5, *p* < 0.001, *ηp*^2^ = 0.87); however, there were no significant weight differences between genotypes (F(2,85) = 1.13, *p* > 0.05, *ηp*^2^ = 0.026) (Figure 2; see Supplementary Figure S1 for body weight over full course of experiment).

### 3.2. Operant Ethanol Self-Administration

**Figure 3:**
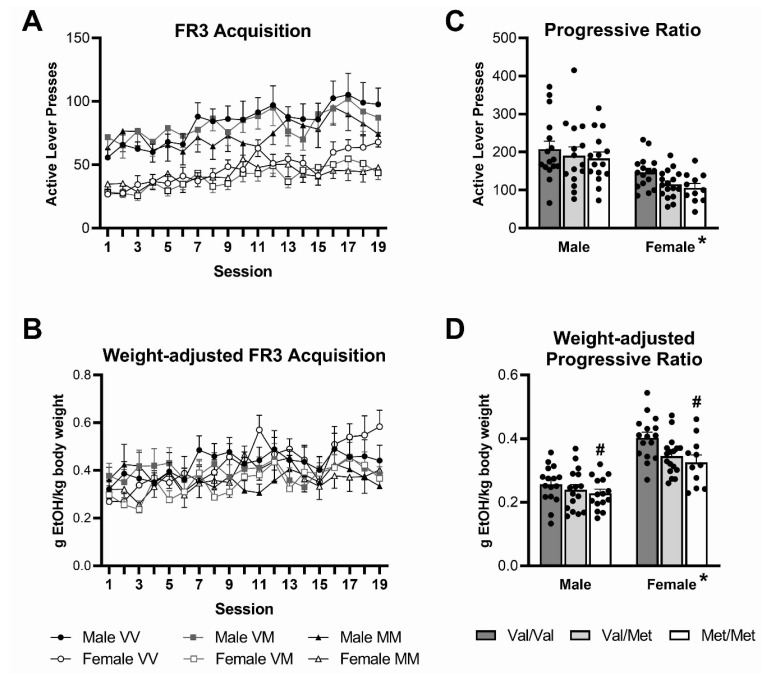
FR3 acquisition: males had significantly higher number of active lever presses for a 10% *v*/*v* ethanol solution than females (**A**); however, there was no difference between the groups in weight-adjusted amount of ethanol ingested (**B**). Progressive ratio analysis: breakpoint number of active lever presses for a 10% *v*/*v* ethanol solution was significantly higher in male compared to female rats (**C**). Weight-adjusted breakpoint amount of ethanol ingested per body weight was significantly higher in female than in male rats (**D**). Met/Met rats had a lower breakpoint than Val/Val rats independent of sex (**D**), but there were no genotype differences in any other measure. Data represent mean ± SEM (n = 11–16 per group). * *p* < 0.001 for difference between males and females, # *p* < 0.05 compared to Val/Val genotype. VV = Val/Val rats; VM = Val/Met rats; MM = Met/Met rats.

All rats successfully learned to operate the levers within the chamber and showed preference for the ethanol and sucrose-paired lever during the overnight training and sucrose fade sessions (see Supplementary Figures S2 and S3). An analysis of baseline acquisition of active ethanol lever pressing for 10% ethanol at FR3 (Figure 3A; see Supplementary Figure S4 for active and inactive lever comparison) showed a main effect for session (F(18,1464) = 12.5, *p* < 0.001, *ηp*^2^ = 0.13), with lever pressing increasing over time. There were no significant interactions between session and either sex or genotype, suggesting that all groups acquired stable responses at the same rate. There was a main effect of sex (F(1,83) = 40.7, *p* < 0.001, *ηp*^2^ = 0.33), with males pressing more than females, but no significant effects of genotype (F(2,83) = 0.46, *p* > 0.05, *ηp*^2^ = 0.011, sex × genotype F(2,83) = 0.25, *p* > 0.05, *ηp*^2^ = 0.006).

To take into account the significant sex differences in body weight, data were also analysed as the ratio of the amount of ethanol ingested by body weight (Figure 3B). There was no longer any significant main effect of sex (F(1,82) = 0.45, *p* > 0.05, *ηp*^2^ = 0.005), and again no significant effects of genotype (genotype F(2,82) = 1.59, *p* > 0.05, *ηp*^2^ = 0.037, sex × genotype F(2,82) = 0.19, *p* > 0.05, *ηp*^2^ = 0.005), suggesting that, despite the significant interactions with session, there were no significant differences between groups on average stable ethanol responding as a ratio of body weight. There was again a main effect of session (F(18,1476) = 4.87, *p* < 0.001, *ηp*^2^ = 0.056) with the amount of ethanol ingested by body weight increasing over time; however, there were now also significant session × sex (F(18,1476) = 2.28, *p* = 0.005, *ηp*^2^ = 0.027) and session × genotype interactions (F(36,1476) = 1.51, *p* = 0.047, *ηp*^2^ = 0.036). Inspection of the data (Figure 3B) suggested that these interactions are due to variable differences between the groups depending on session number, with no consistent trend overall. However, towards the end of the FR3 component of the protocol, the amount of ethanol ingested by body weight was lower in Met/Met rats than in Val/Val rats, although these differences did not reach statistical significance (Figure 3B).

Analysis of the progressive ratio sessions showed that there was no significant main effect of session (F(2,168) = 0.1.19, *p* > 0.05, *ηp*^2^ = 0.014; Supplementary Figure S5A); therefore, further analysis focused on the average of these sessions only (Figure 3C,D; see Supplementary Figure S5B for active and inactive lever comparison). There was a main effect of sex (F(1,84) = 27.9, *p* < 0.001, *ηp*^2^ = 0.25), with males pressing more than females, but no significant effects of genotypes (genotype F(2,84) = 2.13, *p* > 0.05, *ηp*^2^ = 0.048, sex × genotype F(2,84) = 0.15, *p* > 0.05, *ηp*^2^ = 0.004). However, when accounting for body weight (Figure 3D), females had a higher breakpoint than males (F(1,85) = 77.2, *p* < 0.001, *ηp*^2^ = 0.48) and there was also a significant main effect of genotype (F(2,85) = 5.48, *p* = 0.006, *ηp*^2^ = 0.11), although no interaction between sex and genotype (F(2,85) = 1.29, *p* > 0.05, *ηp*^2^ = 0.029). Bonferroni post hoc test showed that Met/Met rats had a significantly lower breakpoint than Val/Val rats when expressed as weight-adjusted ethanol responding (*p* = 0.001).

**Figure 4:**
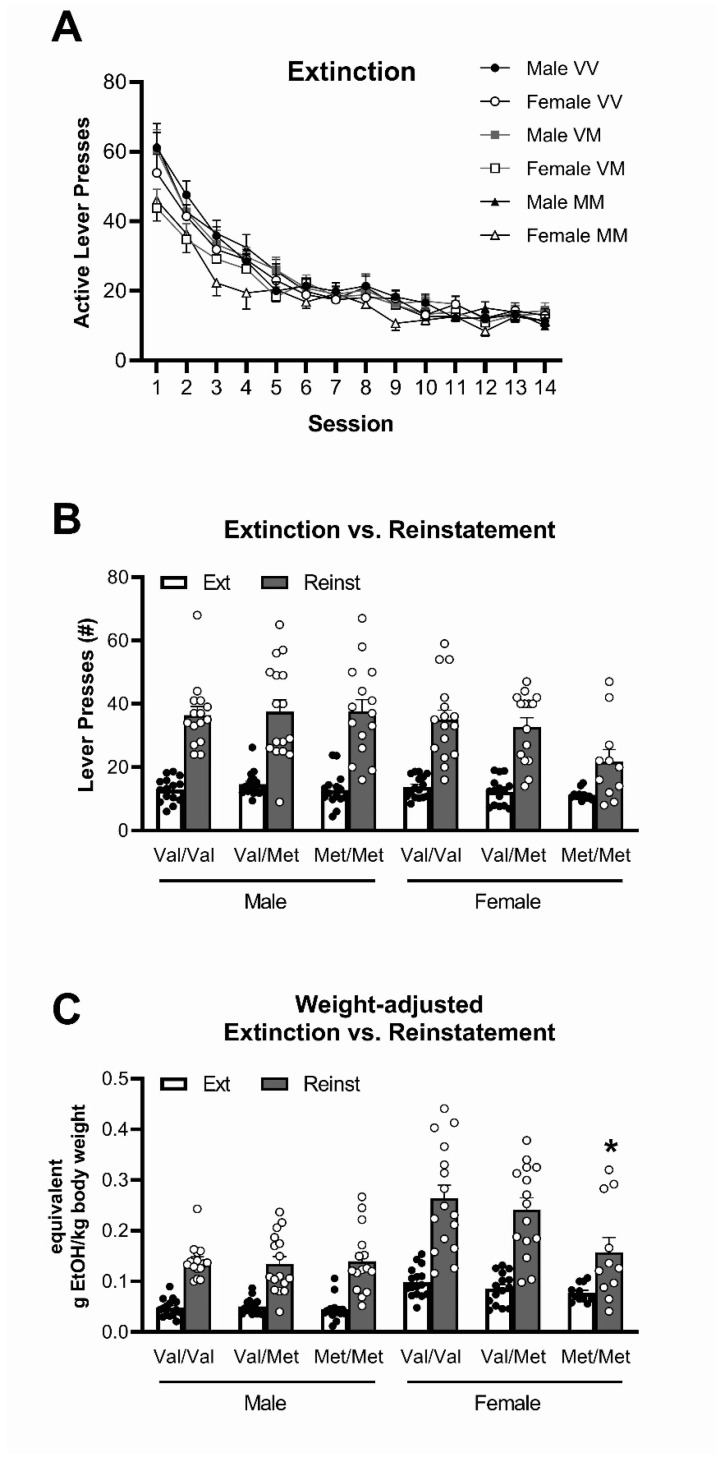
Active lever responding over the 14-day extinction protocol (**A**). Average active lever pressing across the last 5 days of extinction (Ext) vs. the reinstatement (Reinst) session (**B**). Average potential amount of alcohol ingested expressed as ratio of body weight (kg) during the last 5 days of extinction vs. the reinstatement session (**C**). Data represent mean ± SEM (n = 11–16/group). * *p* < 0.05 compared to female Val/Val and Val/Met reinstatement session.

Analysis of extinction sessions following removal of ethanol rewards and cues (Figure 4A) showed a significant effect of session (F(13,1092) = 128.4, *p* < 0.001, *ηp*^2^ = 0.60) as pressing of the previously active lever decreased over time. There was a significant session × sex interaction (F(13,1092) = 3.07, *p* = 0.004, *ηp*^2^ = 0.035), with females starting with lower lever pressing (main effect of sex, F(1,84) = 6.15, *p* = 0.015, *ηp*^2^ = 0.068).

There were no main effects of or interactions with genotype. Analysis of the last 5 days of extinction sessions showed that there were no longer any sex differences for active lever pressing (F(1,84) = 0.66, *p* > 0.05, *ηp*^2^ = 0.008) and no differences between genotypes (genotype F(2,84) = 1.13, *p* > 0.05, *ηp*^2^ = 0.026, sex × genotype F(2,84) = 1.00, *p* > 0.05, *ηp*^2^ = 0.023). It should be noted that during this period, there was still a significant main effect of lever (F(2,84) = 81.2, *p* < 0.001, *ηp*^2^ = 0.50), with lever pressing for the previously active lever being higher than the previously inactive lever (Supplementary Figure S6); however, pressing on both levers decreased over time (Session 1 active 54.8 ± 2.3 presses, inactive 14.6 ± 1.0 presses; Avg last 5 Sessions active 13.2 ± 0.45 presses, inactive 8.6 ± 1.0 presses).

Active lever pressing during a single reinstatement session was compared to the average of the last five sessions of extinction (Figure 4B,C; see Supplementary Figure S7 for active and inactive lever comparison). There was a significant main effect of session (F(1,82) = 253.5, *p* < 0.001, *ηp*^2^ = 0.76), with rats overall showing higher active lever pressing during the reinstatement session compared to the end of extinction. There was also a significant session × sex interaction (F(1,82) = 7.04, *p* = 0.010, *ηp*^2^ = 0.079) although no session × genotype (F(2,82) = 1.38, *p* > 0.05, *ηp*^2^ = 0.032) or session × sex × genotype interaction (F(2,82) = 1.97, *p* > 0.05, *ηp*^2^ = 0.046). However, when taking body weight into account (Figure 4C), while there was again a significant main effect of session (F(1,82) = 211.0, *p* < 0.001, *ηp*^2^ = 0.72) and a significant session × sex interaction (F(1,82) = 7.87, *p* = 0.006, *ηp*^2^ = 0.088), there was also a significant session × sex × genotype interaction (F(2,82) = 3.31, *p* = 0.041, *ηp*^2^ = 0.075). Based on these interactions, body weight-adjusted data were then analysed further with males and females separated. Males showed no significant session × genotype interaction (F(2,43) = 0.20, *p* > 0.05, *ηp*^2^ = 0.009), suggesting that all groups showed the same level of reinstatement. In contrast, there was a large and significant session × genotype interaction in females (F(2,39) = 1.97, *p* = 0.049, *ηp*^2^ = 0.14). Bonferroni post hoc test comparing active lever pressing during extinction and reinstatement showed that Val/Val (*p* < 0.001), Val/Met (*p* < 0.001) as well as Met/Met (*p* = 0.016) rats all showed reinstatement of ethanol responding. However, while there were no genotype differences during extinction sessions, Met/Met females had significantly lower active lever pressing during the reinstatement session compared to both Val/Val (*p* < 0.001) and Val/Met (*p* = 0.012) females (Figure 4C).

Inspection of the data (Figure 4C) suggested differences in the distribution of responding around the mean in female Met/Met rats compared to both Val/Met and Val/Val rats, with some Met/Met rats reinstating their responding similar to the other genotypes, but most of the others responding at the level of the last extinction session. To analyse this, both the number of active lever presses and the weight-adjusted reinstatement data were subjected to Chi-square (χ^2^) analysis. First, a grand mean value was calculated for all female or male rats, and the number of rats of each genotype that were either higher or lower than this grand mean was expressed as percentage (Table 2). This percentage was then compared to a 50/50% chance distribution that would be expected if there were no group differences. The analysis shows that for both number of active lever presses and potential amount of alcohol ingested expressed as ratio of body weight, female Met/Met were significantly different from a 50/50% distribution around the grand mean. Specifically, significantly more rats than expected displayed responses below the expected grand mean level (Table 2, Figure 4B,C).

Analysis of latency to first press the active lever during the reinstatement session showed a significant main effect of sex (F(1,85) = 16.9, *p* < 0.001, *ηp*^2^ = 0.17), with females first pressing later than males. Female Met/Met rats had the longest latencies, but there was no significant main effect of, or interactions with, genotype (Supplementary Figure S8A). Similarly, analysis of latency to first obtain the equivalent of a reward during the reinstatement session showed a significant main effect of sex (F(1,85) = 13.7, *p* < 0.001, *ηp*^2^ = 0.14) with longer latencies in females than males, particularly in female Met/Met rats, but no significant main effect of, or interactions with, genotype (Supplementary Figure S8B).

### 3.3. Elevated plus Maze and Locomotor Activity

**Figure 5:**
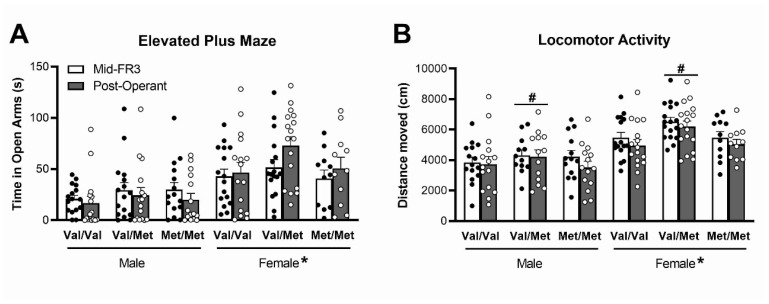
Time spent in the open arms of the elevated plus maze (**A**) and locomotor activity distance moved over 30 min in a photocell chamber (**B**) measured at the mid-point of FR3 acquisition training and following the completion of operant testing. Time spent in open arms increased in females only, while locomotor activity decreased in all groups across sessions. Females had higher time in open arms and locomotor activity than males; Val/Met rats had higher locomotor activity than other genotypes independent of sex. Data represent mean ± SEM (n = 11–16/group). * *p* < 0.001 for difference between males and females, # *p* < 0.05 compared to Val/Val and Met/Met genotype.

Behavioural testing to assess any genotype differences between anxiety-like behaviour or locomotor activity which could affect operant responding was conducted at two time points throughout the testing period. Time spent in the open arm of the elevated plus maze was used as a measure of anxiety-like behaviour (Figure 5A). There was a large main effect of sex (F(1,85) = 27.0, *p* < 0.001, *ηp*^2^ = 0.24), with females spending more time in the open arms than males. There was no main effect of session (F(1,85) = 0.60, *p* > 0.05, *ηp*^2^ = 0.007), suggesting rats explored the open arms of the maze similarly when tested during the middle of the operant protocol and again at the end; however, there was a session × sex interaction (F(1,85) = 6.84, *p* = 0.011, *ηp*^2^ = 0.074). Analysis of males and females separately showed there was no difference in time spent in the open arm across the two testing sessions in males (F(1,44) = 2.25, *p* > 0.05, *ηp*^2^ = 0.049); however, females spent more time in the open arm when tested following completion of operant testing compared to midway through (F(1,41) = 4.47, *p* = 0.041, *ηp*^2^ = 0.098).

Total distance travelled over 30 min in locomotor chambers was measured to determine baseline locomotor activity. There was a main effect of session (F(1,78) = 6.65, *p* = 0.012, *ηp*^2^ = 0.079), with rats less active in the second session, most likely due to habituation to the testing chamber. There were no interactions with sessions, suggesting this effect was consistent across groups. There was a main effect of sex (F(1,78) = 31.4, *p* < 0.001, *ηp*^2^ = 0.29), as females were more active than males, and there was also a main effect of genotype (F(2,78) = 3.67, *p* = 0.030, *ηp*^2^ = 0.086), although no sex × genotype interaction (F(2,78) = 0.48, *p* = 0.62, *ηp*^2^ = 0.012). Bonferroni post hoc test of the genotype effect showed that Val/Met rats were more active than both Val/Val (*p* = 0.014) and Met/Met rats (*p* = 0.016) in the photocell chambers used to measure distance travelled (Figure 5B).

## 4. Discussion

This study showed that, despite no effect of Val68Met genotype in acquisition of stable responding to ethanol or extinction of this response upon removal of reward paired cues, female rats with the Met/Met genotype demonstrate a decreased reinstatement of responding to cues compared to other genotypes, while Met/Met rats of both sexes have a subtly, but significantly lower breakpoint during progressive ratio sessions than Val/Val wildtype rats. These differences were seen primarily when actual or potential amount of alcohol consumed by body weight was taken into account, despite no significant effects of genotype on body weight at any age. There were also no effects of Val68Met genotype on anxiety-like behaviour or locomotor activity which could have affected the way rats behave and respond to lever presentation in the operant self-administration chambers.

The results of this study are consistent with previous work in our laboratory where we have shown sex differences in various animal models of the role of BDNF in operant alcohol self-administration [19,20,31]. In a mouse model of the Val66Met polymorphism, female Val/Val mice showed greater FR3 ethanol self-administration relative to males of the same genotype, while no sex difference was seen in Met/Met mice [31]. Despite this difference, there was no effect of genotype on breakpoint in a progressive ratio session either as total number of lever presses or as of the amount of ethanol ingested by body weight [31]. This contrasts with the current results seen in our rat model where there was no significant effect of genotype during stable ethanol responding, while Met/Met rats showed a slightly, but significantly lower breakpoint than Val/Val wildtype controls, suggesting decreased motivation to continue pressing for an ethanol reward. These results highlight the importance of studying effects of genetics in difference species and genetic models. The mouse model used in our previous study had a human DNA sequence containing the Val66Met polymorphism inserted [37]. The rat model used in the present study carries a valine to methionine substitution at the equivalent position 68 on the BDNF gene [33].

In addition to a difference in breakpoint, we also showed that, after adjusting for body weight, females with the Met/Met genotype selectively showed decreased reinstatement of lever responding following re-presentation of alcohol paired cues. We previously demonstrated that treatment with the TrkB receptor agonist, DHF, increased reinstatement selectively in females, again after adjusting for body weight [20]. As the Met/Met allele is associated with a functional reduction of BDNF release in the brain, the current results appear to fit with these previous findings where enhancing BDNF signalling with DHF increased reinstatement. That is, taken together, the results suggest increased BDNF signalling may lead to increased reinstatement in females, while decreased BDNF signalling appears to have the opposite effect. Both results seem to contradict another previous finding of the laboratory, where female BDNF HET rats displayed significantly higher reinstatement in alcohol responding than female WT controls, with no genotype difference in males [19], as well as studies in BDNF HET mice showing higher baseline ethanol consumption and higher relapse rates compared to controls [16,17,18]. However, it is possible that in these models with severe depletion of endogenous BDNF levels, compensatory changes in the circuitry involved in alcohol self-administration occur, resulting in the animals responding much like after treatment with DHF [38].

That Met/Met rats appear to have lower motivation to continue to press for a reward when the number of presses becomes too high in the progressive ratio sessions, and also show decreased response to return of alcohol-paired cues in the reinstatement session, suggests a possible protective effect of the Met/Met genotype against the likelihood of relapse, at least in females. This is consistent with clinical studies showing higher relapse rates in Val/Val carriers compared to Met allele carriers [29] and that BDNF Val/Val homozygosity confers genetic susceptibility towards substance dependence [25]. However, some mouse studies have suggested the opposite, with greater ethanol consumption seen in Met/Met mice in a two-bottle free-choice paradigm [30]. Other previous studies have suggested that increased BDNF signalling may have a potential protective role in the development of alcohol dependence [9], and alcohol intake has been shown to reduce BDNF expression in rodents [39,40]. Reduced BDNF in several brain regions has also been associated with increased alcohol preference in rodent studies [12,41,42,43]. Alcohol-preferring rats (P rats) show reductions in BDNF mRNA in the medial and central amygdala [10] and BDNF protein in NAc [43], although amygdaloidal BDNF may repress ethanol intake [41]. As the Met/Met genotype leads to decreased activity-dependent release of BDNF, our results appear to contradict this previous research; however the relationship between BDNF levels, Val66Met genotype and alcohol preference may not be simple and may depend on which aspect of alcohol dependence is studied and which methodology is used, for example voluntary intake in a two-bottle choice paradigm vs. operant behaviour; acquisition of intake vs. sustained intake; chronic consumption vs. reinstatement following extinction; targeting specific brain regions vs. systemic treatments which are more clinically relevant. Finally, the concentration of ethanol offered may be important. For example, Warnault et al. [30] found that a Val68Met mouse model showed excessive drinking of a 20% ethanol solution, but not a 10% concentration, which was used in the present study. Future studies should address the influence of these methodological factors on the effect of the Val66Met polymorphism on alcohol seeking and relapse.

The contradictory results between clinical and pre-clinical studies may also reflect limitations in the translatability of findings in some of the rodent models used so far. Two meta analyses have cast doubt on genetic linkage of the Val66Met (rs6265) polymorphism and risk for alcohol dependence [24,25]. This negative result was found even if study populations were stratified according to ethnic background (Caucasian vs. Asian) [24] which is relevant because the frequency of Val66Met markedly differs between ethnic groups [44]. However, as discussed by Tsai [45], even if the BDNF Val66Met polymorphism is not a true genetic risk variant for AUD but links to putative true functional loci with differing strengths among populations, the results from preclinical studies may apply only to some ethnic subgroups [44,45]. In the clinical field, this may be further complicated by possible comorbid psychiatric conditions [32]. In the preclinical field, additional factors of relevance include differences between research groups in background strain of the genetic models used [46], differences in housing and other environmental factors [47], and (as mentioned above) differences in the experimental paradigm used to investigate aspects of alcohol preference and motivation to consume. These results clearly suggest further research is required to determine the role of the Val66Met polymorphism in alcohol dependence and relapse.

The effect of genotype on reinstatement was seen in females only. Female rodents have been extensively shown to have enhanced addiction vulnerability compared to males [48]. Sex differences in the effect of Val66Met in alcohol responding are likely due to the close relationship between BDNF and sex steroid hormones, specifically the ovarian hormone, oestradiol [49]. Expression of BDNF mRNA within the hippocampus and frontal cortex is significantly affected by the increase and decrease in circulating oestradiol levels during the oestrous cycle [50,51,52]. During the oestrous phase, females have been shown to display heightened alcohol-seeking behaviour. Conversely, weakened drug-seeking behaviours were found following ovariectomy [53], with drug seeking returning upon pharmacological oestradiol supplementation [53]. Contrary to this, gonadectomy does not induce decreased self-administration in cocaine in male rats, highlighting importance of the ovarian sex hormone in addiction [54].

Although Val/Met rats were more active than other genotypes, and there were some changes in behaviour as the rats aged and completed more of the operant self-administration protocol, there were no differences in anxiety-like or locomotor activity behaviours between Val/Val wildtype and Met/Met rats. This confirms that the differences in lever pressing between these groups cannot be attributed to baseline differences in anxiety or hyperactivity which could alter the way rats press the lever. These results are mostly consistent with our previous work in this rat model during early adulthood, where we saw no effect of genotype on any measures of anxiety-like behaviour or locomotor activity and only showed decreased fear memory in Met/Met rats compared to other genotypes [34,55].

The subtle increase in exploratory locomotor activity in Val/Met rats compared to both Val/Val and Met/Met genotypes may hint at why Val/Met rats did not differ from Val/Val in any aspect of operant alcohol self-administration. Chen et al. [22] found that the Met/Met genotype was associated with a 29% decrease in BDNF release from mouse hippocampal slices, whereas there was an 18% decrease in BDNF release from Val/Met slices. Neither genotype resulted in altered resting levels of BDNF [22]. The findings on BDNF release would predict that behavioural changes in Val/Met rats would be roughly in between those in Val/Val and Met/Met rats. However, our findings show that behavioural changes do not necessarily follow this gene ‘dosage’ relationship. In a previous study in Val66Met mice [56], we found significantly reduced prepulse inhibition, a model of sensorimotor gating, in Val/Met mice, but not Met/Met mice compared to Val/Val. We reasoned that differential but as-yet-unidentified compensatory mechanisms in Val/Met vs. Met/Met animals may have disrupted the straightforward gene dosage relationship between genotype and behaviour [56]. In the present study, the increased exploratory hyperactivity in Val/Met, but not Met/Met rats, could reflect similar additional behavioural changes in Val/Met rats, not seen in Met/Met rats, which could explain why alcohol seeking and relapse was not different in this genotype compared to Val/Val rats. However, it remains unclear how the slight increase in behavioural activity could mask any changes in progressive ratio breakpoint and reinstatement. Moreover, until these compensatory changes in the brain are identified, this model remains speculative. In BDNF HET mice, we previously showed a significant upregulation of neurotrophin-4 expression in the striatum [57]. It remains to be investigated whether similar changes in the expression of other neurotrophins are found in Met/Met mice. It should be noted that, clinically, it is of utmost relevance that the Val/Met genotype is not necessarily associated with phenotypic changes compared to the Val/Val genotype. In the majority of clinical studies, Val/Val controls are compared to Met ‘carriers’, a group usually consisting mostly of Val/Met individuals (e.g., [58]) because of limited availability of Met/Met individuals when the subjects are from a Western European or North American population [44,45]. Future studies should therefore attempt to include a Met/Met study group of sufficient size to statistically compare all three genotypes.

A limitation of this study is that Met/Met rats may metabolise or excrete alcohol faster than other genotypes, which could have led to lower motivation and reinstatement of alcohol responding. Previously, we found no difference in methamphetamine metabolism between mouse Val66Met genotypes [59], but similar studies have not been performed for alcohol. Further studies should therefore be conducted to measure blood alcohol concentration in these rats. Furthermore, while the present study used an operant paradigm, other measures of alcohol preference, such as a two-bottle free-choice paradigm, could be studied in the Val68Met model to compare to the present results. Finally, future experiments should address whether the changes in motivation we observed, are specific to alcohol. For example, Warnault et al. [30] used a mouse model of Val66Met and showed changes in compulsive alcohol drinking, but not quinine aversion. Jeanblanc et al. [14] showed that infusion of BDNF in the dorsal striatum reduced self-administration of ethanol, but not of sucrose. Future studies in our Val68Met rat model should therefore include self-administration, extinction and reinstatement of other addictive substances for comparison.

## 5. Conclusions

This study showed that in an operant alcohol self-administration paradigm, rats with the Met/Met genotype of the Val66Met polymorphism had a lower breakpoint than Val/Val wildtype rats when the demands to obtain alcohol increase, and that female Met/Met rats had lower propensity for reinstatement to alcohol-paired cues when they were reintroduced following a period of extinction. This suggests that the Met/Met genotype is associated with reduced perseverance and cue-induced relapse of ethanol intake, which is consistent with clinical studies showing increased relapse and susceptibility to dependence in the Val/Val genotype [29]. Despite the caution recommended above, these combined findings would predict that individuals with the Met/Met genotype, particularly females, would be more likely to benefit from psychosocial and/or pharmacological treatments to support abstinence, whereas, conversely, individuals with the Val/Val genotype may need additional support.

## Figures and Tables

**Table 1:** Number of animals per group.

Sex	Genotype	Number
Male	Val/Val	16
	Val/Met	16
	Met/Met	15
Female	Val/Val	16
	Val/Met	17
	Met/Met	11

**Table 2:** Chi square (χ^2^) analysis (with Yates correction) of the proportion of rats in each group above and below the expected grand mean of responding during the reinstatement session. Data are both number of active lever presses and calculated potential amount of alcohol ingested expressed as ratio of body weight. **Bold** indicates groups where significantly more rats than expected displayed responses below the expected grand mean level.

Grand Mean	% Above (n)	% Below (n)	χ^2^	*p*
Female rats—number of active lever presses				
Grand mean = 30.9				
Val/Val	63 (10)	38 (6)	2.644	0.104
Val/Met	63 (10)	38 (6)	2.644	0.104
**Met/Met**	**18 (2)**	**82 (9)**	**21.412**	**<0.001**
Male rats—number of active lever presses				
Grand mean = 37.2				
Val/Val	40 (6)	60 (9)	1.636	0.200
Val/Met	44 (7)	56 (9)	0.502	0.479
Met/Met	40 (6)	60 (9)	1.636	0.200
Female rats—potential amount of alcohol ingested expressed as ratio of body weight				
Grand mean = 0.228				
Val/Val	56 (9)	44 (7)	0.501	0.479
Val/Met	53 (8)	47 (7)	0.080	0.772
**Met/Met**	**27 (3)**	**73 (8)**	**10.221**	**0.001**
Male rats—potential amount of alcohol ingested expressed as ratio of body weight				
Grand mean = 0.138				
Val/Val	40 (6)	60 (9)	1.636	0.200
Val/Met	44 (7)	56 (9)	0.502	0.478
Met/Met	40 (6)	60 (9)	1.636	0.200

## Data Availability

Data is available upon request.

## Supplementary Materials

The following supporting information can be downloaded at: https://www.mdpi.com/article/10.3390/biology12060799/s1.
