# Supplementary material for: A Rat Model of the Brain-Derived Neurotrophic Factor Val66Met Polymorphism Shows Attenuated Motivation for Alcohol Self-Administration and Diminished Propensity for Cue-Induced Relapse in Females
Source: Biology (Basel). 2023 May 31;12(6):799. doi: 10.3390/biology12060799 (PMC10295107; doi:10.3390/biology12060799)
Supplement: Supplementary file 1 [file biology-12-00799-s001.zip › biology-2330457-supplementary.pdf]

A Rat Model of the Brain-Derived Neurotrophic Factor Val66Met Polymorphism Shows Attenuated Motivation for Alcohol Self-Administration and Diminished Propensity for Cue-Induced Relapse in Females

Emily J. Jaehne [1], Elizabeth McInerney [2], Ronan Sharma [1], Shannyn G. Genders [2], Elvan Djouma [2] and Maarten van den Buuse [1,3,*]

[1] Department of Psychology, Counselling and Therapy, School of Psychology and Public Health, La Trobe University, Melbourne 3086, Australia; e.jaehne@latrobe.edu.au (E.J.J.)
[2] Department of Microbiology, Anatomy, Physiology and Pharmacology, School of Agriculture, Biomedicine and Environment, La Trobe University, Melbourne 3086, Australia; e.djouma@latrobe.edu.au (E.D.)
[3] Department of Pharmacology, University of Melbourne, Melbourne 3052, Australia
* Correspondence: m.vandenbuuse@latrobe.edu.au

Supplementary Results

Body weight

Body weight analysed over the course of the experiment showed an expected significant effect of age ($F(15,1260) = 2961$, $p < 0.001$, $\eta p^2 = 0.97$), an age x sex interaction ($F(15,1260) = 497.0$, $p < 0.001$, $\eta p^2 = 0.86$) and a main effect of sex ($F(1,84) = 474.0$, $p < 0.001$, $\eta p^2 = 0.85$), although no significant main effect of, or interactions with, genotype (Supplementary Figure S1).

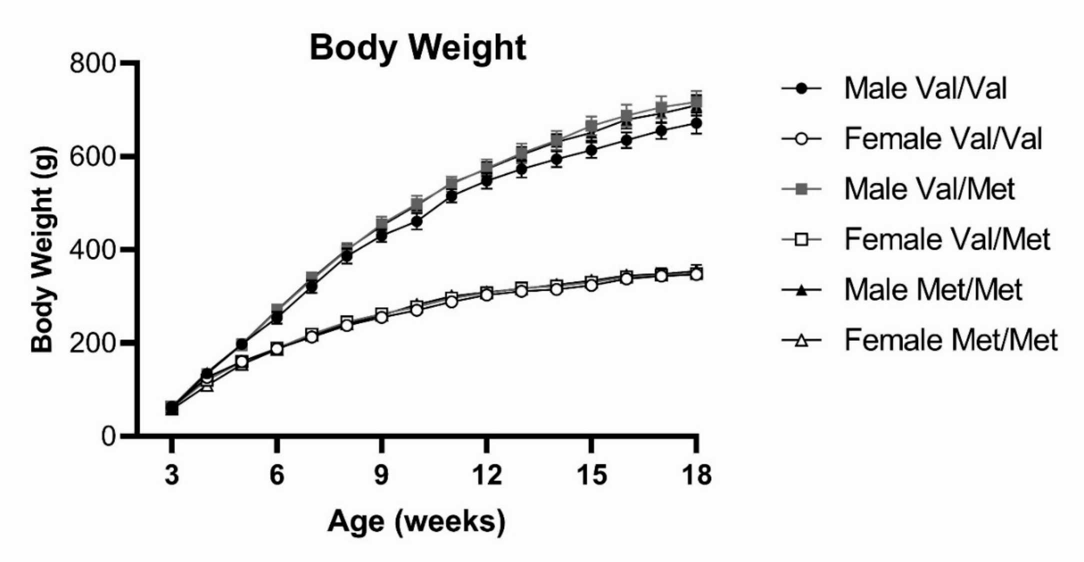

Figure S1. Body weight over the course of the experiment. Males weighed significantly more than females, however there were no significant weight differences between genotypes. Data represent mean ± SEM (n = 11–16/group).

Overnight training

Analysis of lever pressing during the overnight training session showed a significant main effect of lever ($F(1,84) = 72.0$, $p < 0.001$, $\eta p^2 = 0.46$), with rats already showing a preference for the ethanol + sucrose lever. There was also a significant lever x sex interaction ($F(1,84) = 5.72$, $p = 0.019$, $\eta p^2 = 0.064$) and a main effect of sex ($F(1,84) = 8.55$, $p = 0.004$, $\eta p^2 = 0.094$), with females pressing more than males, but no significant main effect of, or interactions with, genotype (Supplementary Figure S2).

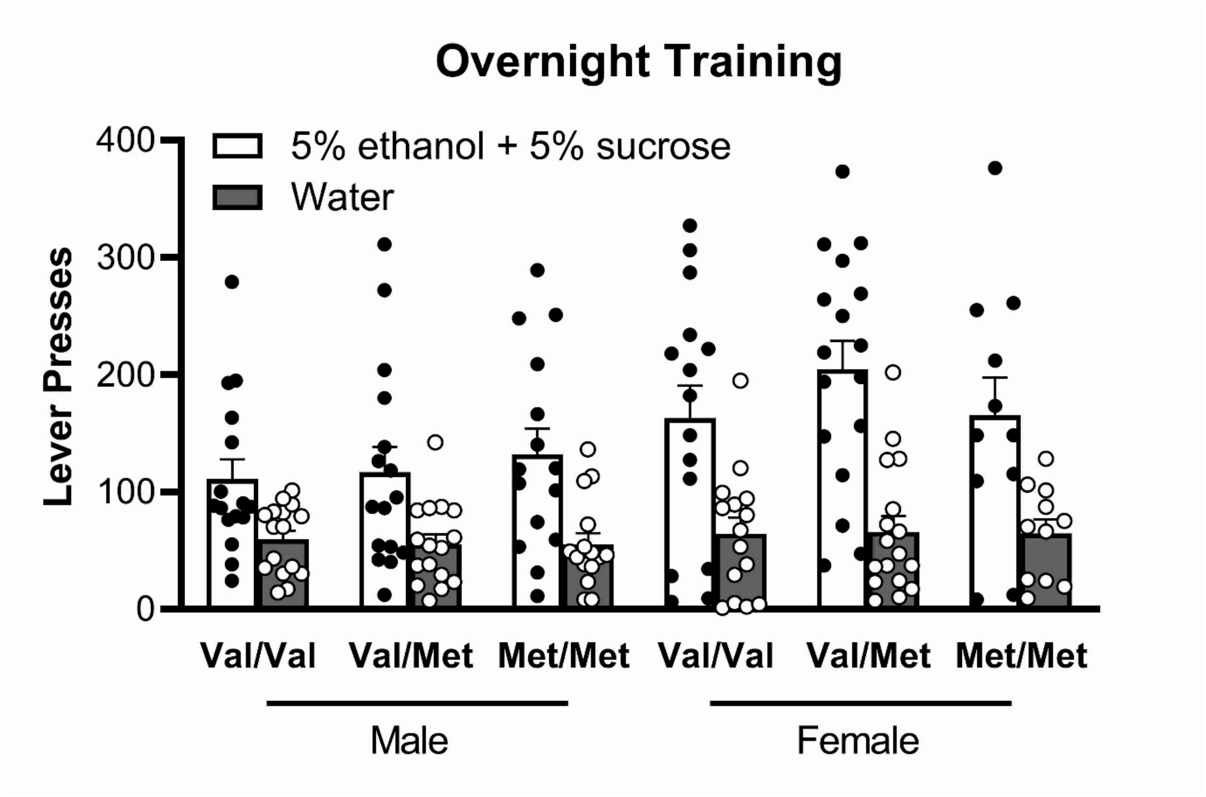

Figure S2. Overnight training: all rats had a significant preference for the ethanol + sucrose lever and females had significantly higher number of total presses than males. Data represent mean ± SEM (n = 11–16/group).

Sucrose Fade

Analysis of the sucrose fade training period showed significant main effects of lever ($F(1,84) = 213.4$, $p < 0.001$, $\eta p^2 = 0.72$), day ($F(7,588) = 40.4$, $p < 0.001$, $\eta p^2 = 0.33$) and sex ($F(1,84) = 27.7$, $p < 0.001$, $\eta p^2 = 0.25$) as well as a significant lever x day interaction ($F(7,588) = 41.2$, $p < 0.001$, $\eta p^2 = 0.33$) and significant interactions between sex, lever and day. These results indicate that rats preferred the ethanol + sucrose lever and that this difference increased over the days, while males pressed more than females. There was no significant main effect of, or interactions with, genotype (Supplementary Figure S3).

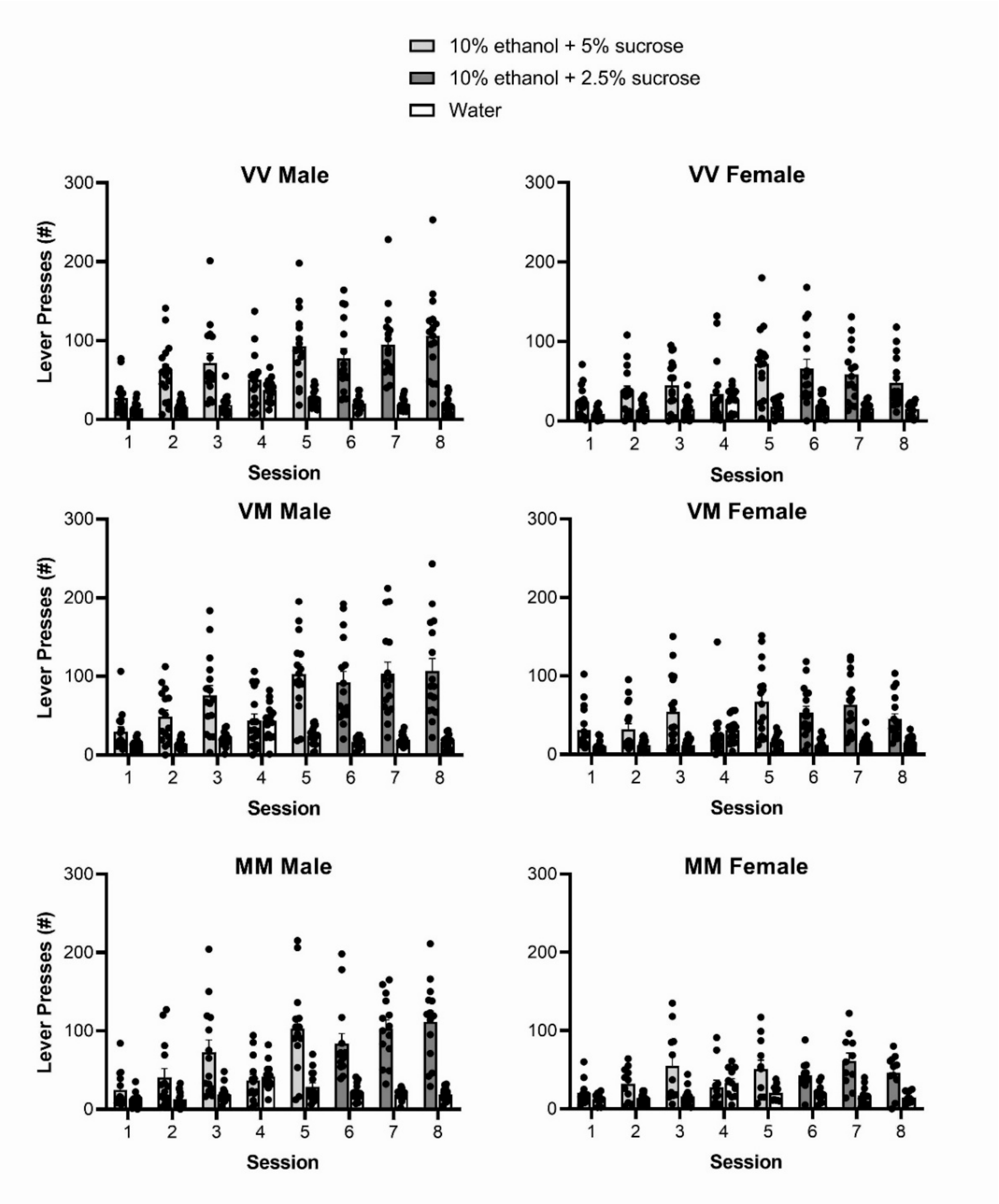

Figure S3. Sucrose Fade: all rats had a significant preference for the ethanol + sucrose lever across the 8 days of sucrose fade training and males pressed more than females. Data represent mean ± SEM (n = 11–16/group). VV = Val/Val rats; VM = Val/Met rats; MM = Met/Met rats.

FR3 lever pressing

Additional analysis of baseline acquisition by lever showed a significant main effect of lever ($F(1,83) = 212.5$, $p < 0.001$, $\eta p^2 = 0.72$), confirming a preference for the ethanol lever, as well as significant lever x sex ($F(1,83) = 30.8$, $p < 0.001$, $\eta p^2 = 0.27$) and lever x day ($F(18,1494) = 13.8$, $p < 0.001$, $\eta p^2 = 0.14$) interactions. Although there was a main effect of sex ($F(1,83) = 45.9$, $p < 0.001$, $\eta p^2 = 0.36$), with males pressing more than females, there was no lever x day x sex interaction and no significant main effect of, or interactions with, genotype (Supplementary Figure S4).

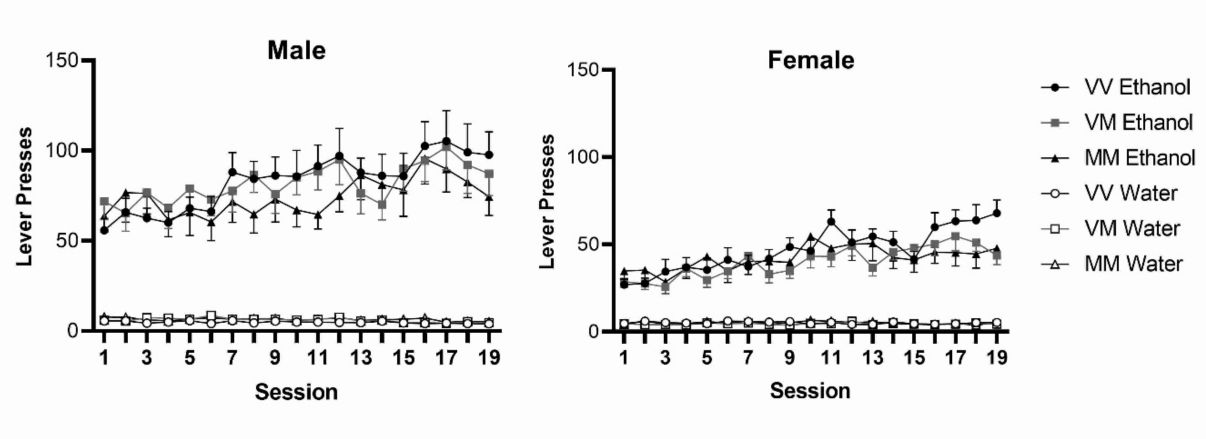

Figure S4. FR3 Acquisition comparison of water and ethanol lever presses: all rats had a significant preference for the ethanol lever and males pressed more than females. Data represent mean ± SEM (n = 11–16/group). VV = Val/Val rats; VM = Val/Met rats; MM = Met/Met rats.

Progressive Ratio

Additional analysis of progressive ratio sessions by lever (Supplementary Figure S5B) showed a significant main effect of lever ($F(1,84) = 256.1$, $p < 0.001$, $\eta p^2 = 0.75$) as well as a significant lever x sex interaction ($F(1,84) = 212.5$, $p < 0.001$, $\eta p^2 = 0.72$). There was no main effect of day ($F(2,168) = 1.48$, $p = 0.23$, $\eta p^2 = 0.017$) and no lever x day interaction ($F(2,168) = 0.86$, $p = 0.41$, $\eta p^2 = 0.010$), showing responding remained consistent over the three sessions conducted (Supplementary Figure S5A). Although there was a main effect of sex ($F(1,84)= 24.0$, $p < 0.001$, $\eta p^2 = 0.22$), with males pressing more than females, there was no significant main effect of, or interactions with, genotype.

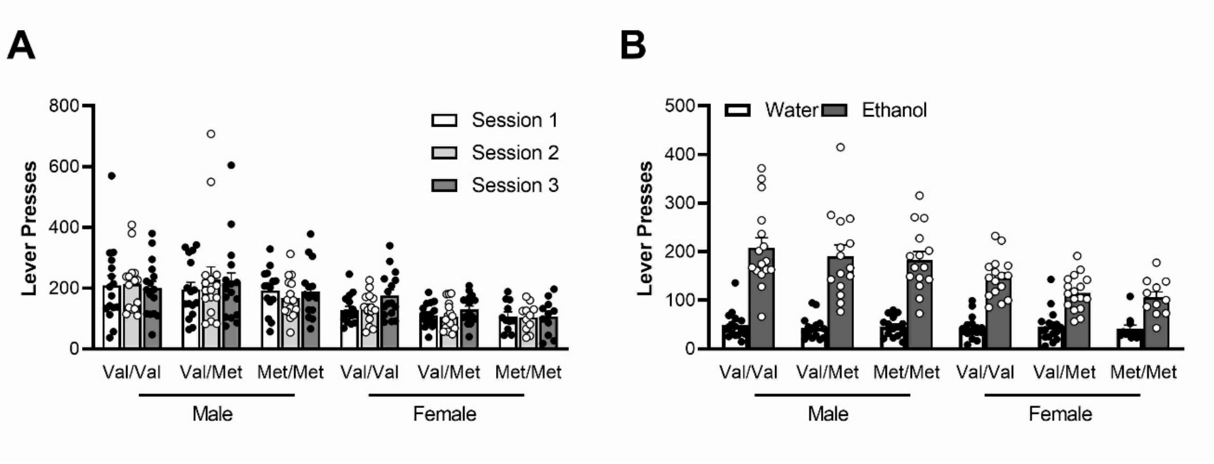

Figure S5. Progressive Ratio active ethanol lever pressing by session (**A**) and comparison of water and ethanol lever presses as an average for all 3 sessions (**B**). There was no difference in active lever pressing between the three repeated sessions. All rats had a significant preference for the ethanol lever and males pressed more than females. Data represent mean ± SEM (n = 11–16/group).

Extinction

Additional analysis of extinction sessions by lever showed a significant main effect of lever ($F(1,83) = 424.5$, $p < 0.001$, $\eta p^2 = 0.84$) as well as a significant lever x day interaction ($F(13,1079) = 85.6$, $p < 0.001$, $\eta p^2 = 0.51$). There was a main effect of sex ($F(1,83) = 8.75$, $p = 0.004$, $\eta p^2 = 0.095$) as well as a lever x day x sex interaction ($F(13,1079) = 2.28$, $p = 0.024$, $\eta p^2 = 0.027$), with males pressing more but decreasing pressing at a faster rate than females. There was no significant main effect of, or interactions with, genotype (Supplementary Figure S6).

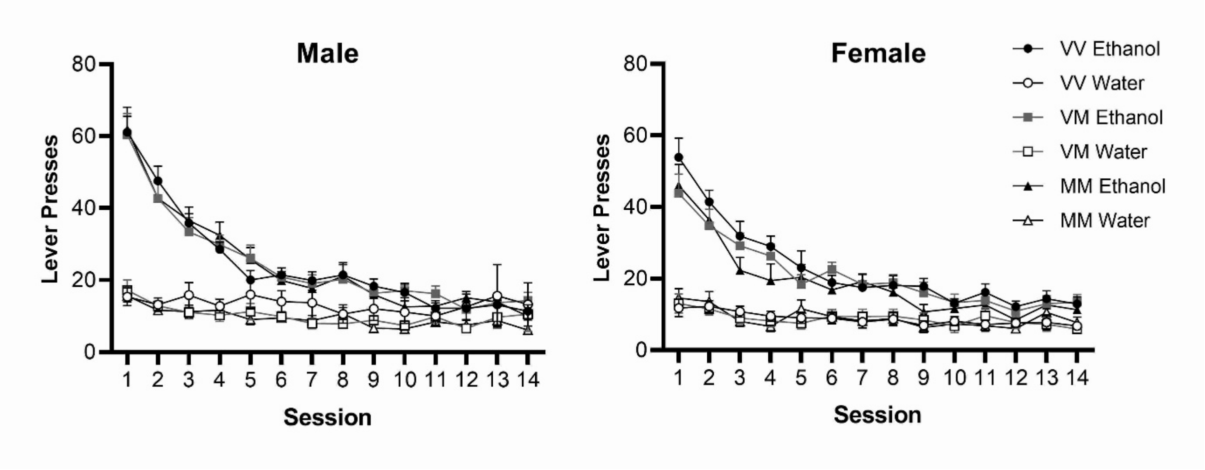

Figure S6. Extinction comparison of previously assigned water and ethanol lever presses: all rats had a significant preference for the ethanol lever which decreased over time and males pressed more than females. Data represent mean ± SEM (n = 11–16/group). VV = Val/Val rats; VM = Val/Met rats; MM = Met/Met rats.

Reinstatement

Analysis of the reinstatement session by lever showed a significant main effect of lever ($F(1,82) = 191.9$, $p < 0.001$, $\eta p^2 = 0.71$). There was also a significant lever x sex interaction ($F(1,82) = 5.76$, $p = 0.019$, $\eta p^2 = 0.066$) and a main effect of sex ($F(1,82) = 4.16$, $p = 0.045$, $\eta p^2 = 0.048$), with females pressing more than males, but no significant main effect of, or interactions with, genotype (Supplementary Figure S7).

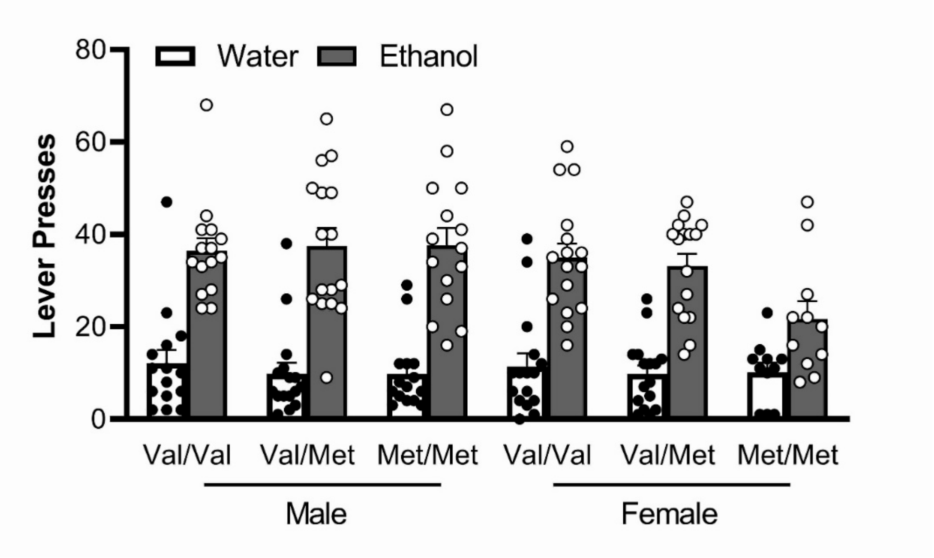

Figure S7. Reinstatement comparison of previously assigned water and ethanol lever presses: all rats had a significant preference for the ethanol lever and males pressed more than females. Data represent mean ± SEM (n = 11–16/group).

Analysis of latency to first press the active lever during the reinstatement session showed a significant main effect of sex ($F(1,85) = 16.9$, $p < 0.001$, $\eta p^2 = 0.17$) with females first pressing later than males. Female Met/Met rats had the longest latencies but there was no significant main effect of, or interactions with, genotype (Supplementary Figure S8A). Similarly, analysis of latency to first obtain the equivalent of a reward during the reinstatement session showed a significant main effect of sex ($F(1,85) = 13.7$, $p < 0.001$, $\eta p^2 = 0.14$) with longer latencies in females than males, particularly in female Met/Met rats, but no significant main effect of, or interactions with, genotype (Supplementary Figure S8B).

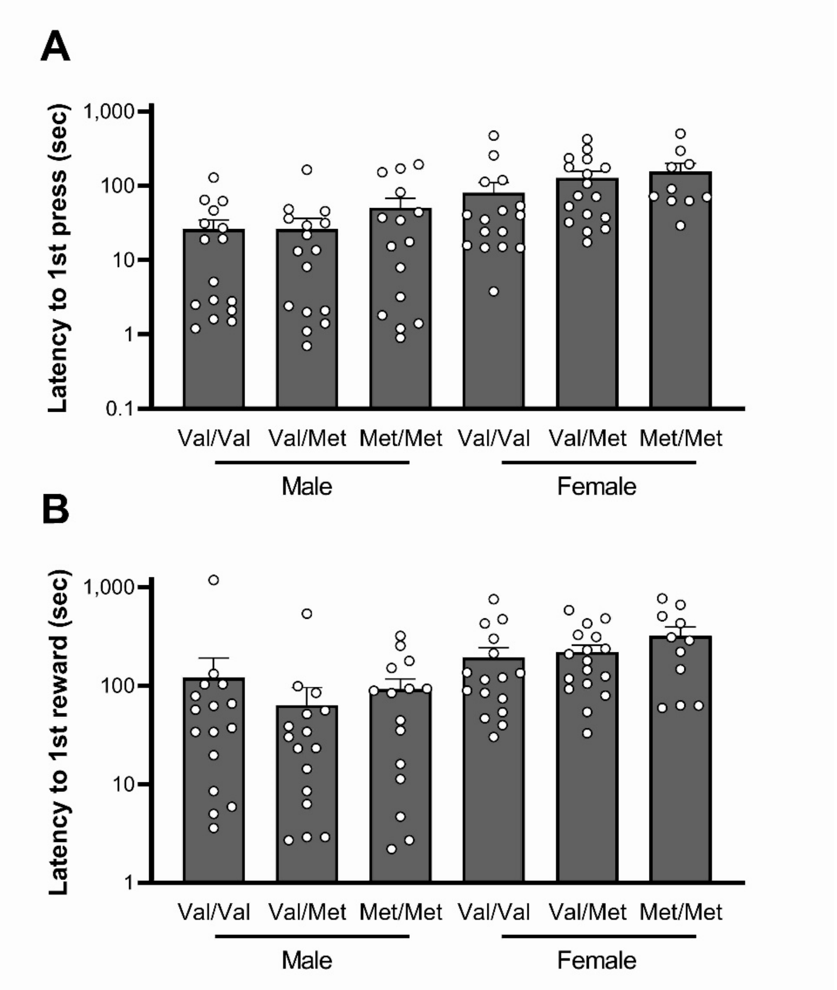

Figure S8. Reinstatement latency comparison of previously assigned ethanol lever presses: females first pressed later than males (**A**) and reached the equivalent of a reward later than males (**B**). To account for variability of the data, the Y-axis was converted to log scale. Data represent mean ± SEM (n = 11–16/group).
